# Supplementary material for: Multi-omics analyses reveal that the gut microbiome and its metabolites promote milk fat synthesis in Zhongdian yak cows
Source: PeerJ. 2022 Dec 2;10:e14444. doi: 10.7717/peerj.14444 (PMC9744170; doi:10.7717/peerj.14444)
Supplement: Supplemental Information 13 [file peerj-10-14444-s013.zip › Web_Report/HTML/meta_kegg_anno.xls.html]

meta\_kegg\_anno.xls


## meta\_kegg\_anno.xls

| #ID | name | KEGG\_annotation | KEGG\_pathway\_annotation |
| --- | --- | --- | --- |
| pos\_1 | d-erythro-dihydrosphingosine | C00836 | Sphingolipid metabolism(ko00600);;Sphingolipid signaling pathway(ko04071);;Metabolic pathways(ko01100) |
| pos\_10 | deoxycholic acid | C04483 | Bile secretion(ko04976) |
| pos\_1005 | bovinic acid | C04056 | Linoleic acid metabolism(ko00591) |
| pos\_1007 | pe(14:0/22:4(7z,10z,13z,16z)) | C00350 | Kaposi sarcoma-associated herpesvirus infection(ko05167);;Glycerophospholipid metabolism(ko00564);;Autophagy - animal(ko04140);;Glycosylphosphatidylinositol (GPI)-anchor biosynthesis(ko00563);;Pathogenic Escherichia coli infection(ko05130);;Retrograde endocannabinoid signaling(ko04723);;Metabolic pathways(ko01100);;Autophagy - other(ko04136) |
| pos\_101 | 8z,11z,14z-heptadecatrienal | C16343 | alpha-Linolenic acid metabolism(ko00592) |
| pos\_1018 | pc(22:2(13z,16z)/22:5(7z,10z,13z,16z,19z)) | C00157 | Retrograde endocannabinoid signaling(ko04723);;Choline metabolism in cancer(ko05231);;alpha-Linolenic acid metabolism(ko00592);;Metabolic pathways(ko01100);;Glycerophospholipid metabolism(ko00564);;Linoleic acid metabolism(ko00591);;Arachidonic acid metabolism(ko00590) |
| pos\_1019 | 20-hydroxy-leukotriene b4 | C04853 | Arachidonic acid metabolism(ko00590) |
| pos\_1022 | nicotinate d-ribonucleoside | C05841 | Metabolic pathways(ko01100);;Nicotinate and nicotinamide metabolism(ko00760) |
| pos\_1025 | deoxyadenosine | C00212 | Purine metabolism(ko00230);;Metabolic pathways(ko01100);;Alcoholism(ko05034);;Parkinson disease(ko05012);;Regulation of lipolysis in adipocytes(ko04923);;Neuroactive ligand-receptor interaction(ko04080);;cGMP-PKG signaling pathway(ko04022);;Renin secretion(ko04924);;Sphingolipid signaling pathway(ko04071);;cAMP signaling pathway(ko04024);;ABC transporters(ko02010);;Morphine addiction(ko05032);;Vascular smooth muscle contraction(ko04270) |
| pos\_1027 | 2-oxoarginine | C03771 | Metabolic pathways(ko01100);;Arginine and proline metabolism(ko00330);;D-Arginine and D-ornithine metabolism(ko00472) |
| pos\_1044 | l-leucine | C00123 | Central carbon metabolism in cancer(ko05230);;Shigellosis(ko05131);;mTOR signaling pathway(ko04150);;Biosynthesis of amino acids(ko01230);;Protein digestion and absorption(ko04974);;Metabolic pathways(ko01100);;2-Oxocarboxylic acid metabolism(ko01210);;Valine, leucine and isoleucine degradation(ko00280);;ABC transporters(ko02010);;Aminoacyl-tRNA biosynthesis(ko00970);;Mineral absorption(ko04978);;Valine, leucine and isoleucine biosynthesis(ko00290) |
| pos\_1048 | l-threonine | C00188 | Glycine, serine and threonine metabolism(ko00260);;Valine, leucine and isoleucine biosynthesis(ko00290);;Mineral absorption(ko04978);;ABC transporters(ko02010);;Porphyrin and chlorophyll metabolism(ko00860);;Aminoacyl-tRNA biosynthesis(ko00970);;Protein digestion and absorption(ko04974);;Metabolic pathways(ko01100);;Biosynthesis of amino acids(ko01230);;Monobactam biosynthesis(ko00261) |
| pos\_1050 | brassinolide | C08814 | Metabolic pathways(ko01100) |
| pos\_1054 | farnesylcysteine | C19691 | Terpenoid backbone biosynthesis(ko00900) |
| pos\_1087 | galactosylglycerol | C05401 | Glycerolipid metabolism(ko00561);;Galactose metabolism(ko00052) |
| pos\_1110 | 6-keto-prostaglandin f1a | C05961 | Metabolic pathways(ko01100);;Arachidonic acid metabolism(ko00590) |
| pos\_112 | hyacinthin | C00601 | Phenylalanine metabolism(ko00360);;Metabolic pathways(ko01100) |
| pos\_1129 | octanoylglucuronide | C03033 | Bile secretion(ko04976);;Pentose and glucuronate interconversions(ko00040);;Metabolic pathways(ko01100) |
| pos\_1148 | corticosterone | C02140 | Steroid hormone biosynthesis(ko00140);;Regulation of lipolysis in adipocytes(ko04923);;Aldosterone synthesis and secretion(ko04925);;Prion disease(ko05020);;Metabolic pathways(ko01100) |
| pos\_1165 | estradiol | C00951 | Endocrine resistance(ko01522);;GnRH secretion(ko04929);;Steroid hormone biosynthesis(ko00140);;Breast cancer(ko05224);;Endocrine and other factor-regulated calcium reabsorption(ko04961);;Estrogen signaling pathway(ko04915);;Prostate cancer(ko05215);;Thermogenesis(ko04714);;Metabolic pathways(ko01100);;Prolactin signaling pathway(ko04917);;Ovarian steroidogenesis(ko04913);;Pathways in cancer(ko05200) |
| pos\_1169 | testosterone | C00535 | Steroid hormone biosynthesis(ko00140);;Ovarian steroidogenesis(ko04913);;Pathways in cancer(ko05200);;Prostate cancer(ko05215);;Endocrine resistance(ko01522);;GnRH secretion(ko04929);;Metabolic pathways(ko01100) |
| pos\_1181 | 4-chlorocatechol | C02375 | Metabolic pathways(ko01100) |
| pos\_1195 | 3-succinoylpyridine | C19569 | Chemical carcinogenesis(ko05204);;Metabolism of xenobiotics by cytochrome P450(ko00980);;Nicotinate and nicotinamide metabolism(ko00760);;Metabolic pathways(ko01100) |
| pos\_12 | hypoxanthine | C00262 | Purine metabolism(ko00230);;Metabolic pathways(ko01100) |
| pos\_1204 | androsterone | C00523 | Steroid hormone biosynthesis(ko00140) |
| pos\_1241 | acarbose | C06802 | Metabolic pathways(ko01100) |
| pos\_1252 | docosahexaenoic acid | C06429 | Biosynthesis of unsaturated fatty acids(ko01040) |
| pos\_126 | 4-coumaroylshikimate | C02947 | Metabolic pathways(ko01100) |
| pos\_1260 | vitamin a2 | C21797 | Retinol metabolism(ko00830) |
| pos\_1264 | 1-methylhistidine | C01152 | Metabolic pathways(ko01100);;Histidine metabolism(ko00340) |
| pos\_1267 | l-histidine | C00135 | Histidine metabolism(ko00340);;beta-Alanine metabolism(ko00410);;Aminoacyl-tRNA biosynthesis(ko00970);;ABC transporters(ko02010);;Metabolic pathways(ko01100);;Protein digestion and absorption(ko04974);;Biosynthesis of amino acids(ko01230);;Central carbon metabolism in cancer(ko05230) |
| pos\_1275 | l-histidinol | C00860 | Histidine metabolism(ko00340);;Metabolic pathways(ko01100);;Biosynthesis of amino acids(ko01230) |
| pos\_1282 | dolichyl diphosphate | C00621 | N-Glycan biosynthesis(ko00510);;Metabolic pathways(ko01100) |
| pos\_1286 | l-urobilinogen | C05789 | Porphyrin and chlorophyll metabolism(ko00860) |
| pos\_13 | guanosine | C00387 | Metabolic pathways(ko01100);;Purine metabolism(ko00230);;ABC transporters(ko02010) |
| pos\_1315 | isopropylmaleate | C02631 | 2-Oxocarboxylic acid metabolism(ko01210);;Valine, leucine and isoleucine biosynthesis(ko00290) |
| pos\_1355 | stearic acid | C01530 | Phototransduction - fly(ko04745);;Biosynthesis of unsaturated fatty acids(ko01040);;Fatty acid biosynthesis(ko00061) |
| pos\_1362 | sparfloxacin | C07662 | ABC transporters(ko02010) |
| pos\_1370 | fmnh2 | C01847 | Metabolic pathways(ko01100);;Riboflavin metabolism(ko00740) |
| pos\_1383 | 3-hexaprenyl-4-hydroxybenzoic acid | C13425 | Ubiquinone and other terpenoid-quinone biosynthesis(ko00130) |
| pos\_14 | decanoic acid | C01571 | Metabolic pathways(ko01100);;Fatty acid biosynthesis(ko00061) |
| pos\_1407 | 21-deoxycortisol | C05497 | Steroid hormone biosynthesis(ko00140) |
| pos\_1422 | ciprofloxacin | C05349 | ABC transporters(ko02010) |
| pos\_1430 | pc(20:4(5z,8z,11z,14z)/18:0) | C00157 | Arachidonic acid metabolism(ko00590);;Linoleic acid metabolism(ko00591);;Glycerophospholipid metabolism(ko00564);;Metabolic pathways(ko01100);;alpha-Linolenic acid metabolism(ko00592);;Choline metabolism in cancer(ko05231);;Retrograde endocannabinoid signaling(ko04723) |
| pos\_1442 | dg(16:0/18:1(11z)/0:0) | C00165 | Th17 cell differentiation(ko04659);;Regulation of lipolysis in adipocytes(ko04923);;Thyroid hormone signaling pathway(ko04919);;Axon regeneration(ko04361);;Th1 and Th2 cell differentiation(ko04658);;Relaxin signaling pathway(ko04926);;Gastric acid secretion(ko04971);;Insulin resistance(ko04931);;Pathways in cancer(ko05200);;Neurotrophin signaling pathway(ko04722);;Phospholipase D signaling pathway(ko04072);;African trypanosomiasis(ko05143);;Natural killer cell mediated cytotoxicity(ko04650);;Dopaminergic synapse(ko04728);;Glioma(ko05214);;Fc gamma R-mediated phagocytosis(ko04666);;NF-kappa B signaling pathway(ko04064);;Platelet activation(ko04611);;Amoebiasis(ko05146);;PD-L1 expression and PD-1 checkpoint pathway in cancer(ko05235);;Phototransduction - fly(ko04745);;Spinocerebellar ataxia(ko05017);;Vibrio cholerae infection(ko05110);;Vascular smooth muscle contraction(ko04270);;Growth hormone synthesis, secretion and action(ko04935);;Hepatocellular carcinoma(ko05225);;Oxytocin signaling pathway(ko04921);;Carbohydrate digestion and absorption(ko04973);;GnRH signaling pathway(ko04912);;Fat digestion and absorption(ko04975);;Human cytomegalovirus infection(ko05163);;Glutamatergic synapse(ko04724);;Parathyroid hormone synthesis, secretion and action(ko04928);;MAPK signaling pathway(ko04010);;C-type lectin receptor signaling pathway(ko04625);;Human immunodeficiency virus 1 infection(ko05170);;Calcium signaling pathway(ko04020);;Circadian entrainment(ko04713);;VEGF signaling pathway(ko04370);;cAMP signaling pathway(ko04024);;Kaposi sarcoma-associated herpesvirus infection(ko05167);;Sphingolipid signaling pathway(ko04071);;Long-term depression(ko04730);;Parkinson disease(ko05012);;Shigellosis(ko05131);;Inflammatory mediator regulation of TRP channels(ko04750);;Choline metabolism in cancer(ko05231);;Thermogenesis(ko04714);;AGE-RAGE signaling pathway in diabetic complications(ko04933);;Fc epsilon RI signaling pathway(ko04664);;GnRH secretion(ko04929);;Adipocytokine signaling pathway(ko04920);;Chemokine signaling pathway(ko04062);;Serotonergic synapse(ko04726);;Ras signaling pathway(ko04014);;ErbB signaling pathway(ko04012);;Insulin secretion(ko04911);;Pancreatic secretion(ko04972);;Salivary secretion(ko04970);;Retrograde endocannabinoid signaling(ko04723);;Estrogen signaling pathway(ko04915);;Long-term potentiation(ko04720);;Endocrine and other factor-regulated calcium reabsorption(ko04961);;Melanogenesis(ko04916);;Apelin signaling pathway(ko04371);;HIF-1 signaling pathway(ko04066);;Gap junction(ko04540);;EGFR tyrosine kinase inhibitor resistance(ko01521);;Non-small cell lung cancer(ko05223);;Rap1 signaling pathway(ko04015);;B cell receptor signaling pathway(ko04662);;T cell receptor signaling pathway(ko04660);;Thyroid hormone synthesis(ko04918);;Adrenergic signaling in cardiomyocytes(ko04261);;Cholinergic synapse(ko04725);;Aldosterone synthesis and secretion(ko04925) |
| pos\_15 | guanine | C00242 | Purine metabolism(ko00230);;Metabolic pathways(ko01100) |
| pos\_1517 | dethiobiotin | C01909 | Metabolic pathways(ko01100);;Biotin metabolism(ko00780) |
| pos\_152 | biliverdin ix | C00500 | Porphyrin and chlorophyll metabolism(ko00860);;Metabolic pathways(ko01100) |
| pos\_1539 | alpha-fluoro-beta-ureidopropionic acid | C16631 | Drug metabolism - other enzymes(ko00983) |
| pos\_1541 | tryptophanol | C00955 | Tryptophan metabolism(ko00380) |
| pos\_1569 | propoxyphene | C07481 | Caffeine metabolism(ko00232);;Metabolic pathways(ko01100) |
| pos\_1573 | nicotinuric acid | C05380 | Nicotinate and nicotinamide metabolism(ko00760) |
| pos\_16 | rosmarinic acid | C01850 | Metabolic pathways(ko01100);;Tyrosine metabolism(ko00350) |
| pos\_1607 | naringenin | C00509 | Metabolic pathways(ko01100) |
| pos\_161 | 17-hydroxylinolenic acid | C16346 | alpha-Linolenic acid metabolism(ko00592) |
| pos\_1619 | lysopc(20:0) | C04230 | Glycerophospholipid metabolism(ko00564);;Choline metabolism in cancer(ko05231) |
| pos\_1652 | (7z,10z,13z,16z)-docosatetraenoate | C16527 | Biosynthesis of unsaturated fatty acids(ko01040);;Ferroptosis(ko04216) |
| pos\_1674 | (3s)-3,6-diaminohexanoate | C01142 | Lysine degradation(ko00310);;Metabolic pathways(ko01100) |
| pos\_1675 | 3-sulfinato-l-alaninate | C00606 | Cysteine and methionine metabolism(ko00270);;Taurine and hypotaurine metabolism(ko00430);;Metabolic pathways(ko01100) |
| pos\_1697 | moxifloxacin | C07663 | ABC transporters(ko02010) |
| pos\_1705 | 11,14,17-eicosatrienoic acid | C16522 | Biosynthesis of unsaturated fatty acids(ko01040) |
| pos\_1709 | sm(d18:1/18:1(11z)) | C00550 | Metabolic pathways(ko01100);;Sphingolipid signaling pathway(ko04071);;Sphingolipid metabolism(ko00600);;Necroptosis(ko04217) |
| pos\_1714 | arachidonic acid | C00219 | Ferroptosis(ko04216);;Inflammatory mediator regulation of TRP channels(ko04750);;Linoleic acid metabolism(ko00591);;Ovarian steroidogenesis(ko04913);;Retrograde endocannabinoid signaling(ko04723);;Platelet activation(ko04611);;Fc gamma R-mediated phagocytosis(ko04666);;Arachidonic acid metabolism(ko00590);;Amoebiasis(ko05146);;Necroptosis(ko04217);;Phototransduction - fly(ko04745);;Leishmaniasis(ko05140);;Biosynthesis of unsaturated fatty acids(ko01040);;Regulation of lipolysis in adipocytes(ko04923);;Long-term depression(ko04730);;Serotonergic synapse(ko04726);;Aldosterone synthesis and secretion(ko04925);;Metabolic pathways(ko01100);;Oxytocin signaling pathway(ko04921);;Fc epsilon RI signaling pathway(ko04664);;Vascular smooth muscle contraction(ko04270);;GnRH signaling pathway(ko04912) |
| pos\_1717 | 2-isopropylmalic acid | C02504 | 2-Oxocarboxylic acid metabolism(ko01210);;Biosynthesis of amino acids(ko01230);;Pyruvate metabolism(ko00620);;Metabolic pathways(ko01100);;Valine, leucine and isoleucine biosynthesis(ko00290) |
| pos\_1740 | thiamine monophosphate | C01081 | Thiamine metabolism(ko00730);;Metabolic pathways(ko01100) |
| pos\_1745 | cysteamine | C01678 | Metabolic pathways(ko01100);;Taurine and hypotaurine metabolism(ko00430) |
| pos\_1746 | streptomycin | C00413 | Metabolic pathways(ko01100) |
| pos\_1752 | alpha-tocotrienol | C14153 | Ubiquinone and other terpenoid-quinone biosynthesis(ko00130);;Metabolic pathways(ko01100) |
| pos\_1783 | farnesyl pyrophosphate | C00448 | Metabolic pathways(ko01100);;Steroid biosynthesis(ko00100);;Terpenoid backbone biosynthesis(ko00900);;Insect hormone biosynthesis(ko00981) |
| pos\_18 | l-homocystine | C01817 | Cysteine and methionine metabolism(ko00270) |
| pos\_1806 | pc(22:5(4z,7z,10z,13z,16z)/24:1(15z)) | C00157 | Glycerophospholipid metabolism(ko00564);;Metabolic pathways(ko01100);;alpha-Linolenic acid metabolism(ko00592);;Choline metabolism in cancer(ko05231);;Retrograde endocannabinoid signaling(ko04723);;Arachidonic acid metabolism(ko00590);;Linoleic acid metabolism(ko00591) |
| pos\_181 | 1-nitronaphthalene | C14040 | Metabolism of xenobiotics by cytochrome P450(ko00980) |
| pos\_1810 | deoxyadenosine monophosphate | C00360 | Metabolic pathways(ko01100);;Purine metabolism(ko00230) |
| pos\_1817 | 3,4-dihydroxymandelic acid | C05580 | Metabolic pathways(ko01100);;Tyrosine metabolism(ko00350) |
| pos\_1833 | pc(22:5(4z,7z,10z,13z,16z)/22:6(4z,7z,10z,13z,16z,19z)) | C00157 | Linoleic acid metabolism(ko00591);;Arachidonic acid metabolism(ko00590);;Metabolic pathways(ko01100);;Glycerophospholipid metabolism(ko00564);;Retrograde endocannabinoid signaling(ko04723);;Choline metabolism in cancer(ko05231);;alpha-Linolenic acid metabolism(ko00592) |
| pos\_1835 | calcidiol | C01561 | Parathyroid hormone synthesis, secretion and action(ko04928);;Metabolic pathways(ko01100);;Steroid biosynthesis(ko00100);;Tuberculosis(ko05152) |
| pos\_1844 | vitamin k1 | C02059 | Ubiquinone and other terpenoid-quinone biosynthesis(ko00130);;Metabolic pathways(ko01100);;Vitamin digestion and absorption(ko04977) |
| pos\_1860 | dgdp | C00361 | Purine metabolism(ko00230);;Metabolic pathways(ko01100) |
| pos\_1898 | ps(18:0/20:0) | C02737 | Amoebiasis(ko05146);;Leishmaniasis(ko05140);;Glycine, serine and threonine metabolism(ko00260);;Metabolic pathways(ko01100);;Systemic lupus erythematosus(ko05322);;Glycerophospholipid metabolism(ko00564) |
| pos\_1899 | ergothioneine | C05570 | Metabolic pathways(ko01100);;Histidine metabolism(ko00340) |
| pos\_19 | cellobiose | C00185 | Starch and sucrose metabolism(ko00500);;Metabolic pathways(ko01100);;ABC transporters(ko02010) |
| pos\_1904 | linoleic acid | C01595 | Biosynthesis of unsaturated fatty acids(ko01040);;Metabolic pathways(ko01100);;Linoleic acid metabolism(ko00591) |
| pos\_1955 | hippuric acid | C01586 | Metabolic pathways(ko01100);;Phenylalanine metabolism(ko00360) |
| pos\_1980 | 9(s)-hpode | C14827 | Linoleic acid metabolism(ko00591);;Metabolic pathways(ko01100) |
| pos\_1982 | prostaglandin e2 | C00584 | Inflammatory mediator regulation of TRP channels(ko04750);;Leishmaniasis(ko05140);;Regulation of lipolysis in adipocytes(ko04923);;Neuroactive ligand-receptor interaction(ko04080);;cAMP signaling pathway(ko04024);;Arachidonic acid metabolism(ko00590);;Amoebiasis(ko05146);;Rheumatoid arthritis(ko05323);;Bile secretion(ko04976);;C-type lectin receptor signaling pathway(ko04625);;Metabolic pathways(ko01100);;Serotonergic synapse(ko04726);;African trypanosomiasis(ko05143);;Human cytomegalovirus infection(ko05163);;Pathways in cancer(ko05200);;Human papillomavirus infection(ko05165);;Renin secretion(ko04924);;Oxytocin signaling pathway(ko04921) |
| pos\_1986 | dihydrobiopterin | C02953 | Folate biosynthesis(ko00790);;Metabolic pathways(ko01100) |
| pos\_2 | betaine | C00719 | ABC transporters(ko02010);;Metabolic pathways(ko01100);;Glycine, serine and threonine metabolism(ko00260) |
| pos\_2008 | 2,2-dichloro-1,1-ethanediol | C14860 | Metabolism of xenobiotics by cytochrome P450(ko00980) |
| pos\_2048 | phenylacetaldehyde | C00601 | Phenylalanine metabolism(ko00360);;Metabolic pathways(ko01100) |
| pos\_2054 | desmosterol | C01802 | Metabolic pathways(ko01100);;Steroid biosynthesis(ko00100) |
| pos\_2062 | piperidine | C01746 | Protein digestion and absorption(ko04974) |
| pos\_2072 | 2-hydroxyestradiol | C05301 | Steroid hormone biosynthesis(ko00140);;Ovarian steroidogenesis(ko04913) |
| pos\_2105 | glycine | C00037 | Phosphonate and phosphinate metabolism(ko00440);;Primary bile acid biosynthesis(ko00120);;Lysine degradation(ko00310);;Biosynthesis of amino acids(ko01230);;Metabolic pathways(ko01100);;Glyoxylate and dicarboxylate metabolism(ko00630);;Thiamine metabolism(ko00730);;Mineral absorption(ko04978);;Central carbon metabolism in cancer(ko05230);;Purine metabolism(ko00230);;Protein digestion and absorption(ko04974);;Synaptic vesicle cycle(ko04721);;Aminoacyl-tRNA biosynthesis(ko00970);;Porphyrin and chlorophyll metabolism(ko00860);;Glutathione metabolism(ko00480);;ABC transporters(ko02010);;Carbon metabolism(ko01200);;Neuroactive ligand-receptor interaction(ko04080);;Glycine, serine and threonine metabolism(ko00260) |
| pos\_212 | cytidine | C00475 | Metabolic pathways(ko01100);;Pyrimidine metabolism(ko00240);;ABC transporters(ko02010) |
| pos\_2135 | aldosterone 18-glucuronide | C03033 | Bile secretion(ko04976) |
| pos\_2166 | prostaglandin a2 | C05953 | Metabolic pathways(ko01100);;Serotonergic synapse(ko04726);;Arachidonic acid metabolism(ko00590) |
| pos\_2191 | glycinexylidide | C16569 | Drug metabolism - cytochrome P450(ko00982) |
| pos\_220 | retinaldehyde | C00376 | Retinol metabolism(ko00830);;Phototransduction(ko04744);;Metabolic pathways(ko01100);;Vitamin digestion and absorption(ko04977) |
| pos\_2204 | diethanolamine | C06772 | Glycerophospholipid metabolism(ko00564) |
| pos\_2218 | bilirubin diglucuronide | C05787 | Metabolic pathways(ko01100);;Porphyrin and chlorophyll metabolism(ko00860) |
| pos\_2263 | lysopc(20:2(11z,14z)) | C04230 | Glycerophospholipid metabolism(ko00564);;Choline metabolism in cancer(ko05231) |
| pos\_2274 | n-acetylornithine | C00437 | Arginine biosynthesis(ko00220);;Metabolic pathways(ko01100);;Biosynthesis of amino acids(ko01230);;2-Oxocarboxylic acid metabolism(ko01210) |
| pos\_2279 | hydrocinnamic acid | C05629 | Metabolic pathways(ko01100);;Phenylalanine metabolism(ko00360) |
| pos\_2283 | citalopram aldehyde | C16612 | Drug metabolism - cytochrome P450(ko00982) |
| pos\_2328 | guanosine 3'-diphosphate 5'-triphosphate | C04494 | Purine metabolism(ko00230);;Metabolic pathways(ko01100) |
| pos\_2330 | 7,8-dihydro-7-hydroxy-8-s-glutathionyl-benzo[a]pyrene | C14856 | Chemical carcinogenesis(ko05204);;Metabolism of xenobiotics by cytochrome P450(ko00980) |
| pos\_2406 | p1,p4-bis(5'-uridyl) tetraphosphate | C06198 | Metabolic pathways(ko01100);;Pyrimidine metabolism(ko00240) |
| pos\_2409 | cdp-glucose | C00501 | Amino sugar and nucleotide sugar metabolism(ko00520);;Metabolic pathways(ko01100);;Starch and sucrose metabolism(ko00500) |
| pos\_2413 | 5-hydroxyindoleacetaldehyde | C05634 | Metabolic pathways(ko01100);;Tryptophan metabolism(ko00380) |
| pos\_2433 | 2-methylpropyl glucosinolate | C17256 | 2-Oxocarboxylic acid metabolism(ko01210) |
| pos\_2463 | 5-(3-pyridyl)-2-hydroxytetrahydrofuran | C19578 | Metabolism of xenobiotics by cytochrome P450(ko00980);;Chemical carcinogenesis(ko05204) |
| pos\_2465 | 2-propyl-2,4-pentadienoic acid | C16656 | Drug metabolism - cytochrome P450(ko00982) |
| pos\_2466 | delta-tocopherol | C14151 | Ubiquinone and other terpenoid-quinone biosynthesis(ko00130);;Metabolic pathways(ko01100) |
| pos\_2493 | aflatoxin b2 | C16753 | Metabolic pathways(ko01100) |
| pos\_2509 | 1-nitro-5-hydroxy-6-glutathionyl-5,6-dihydronaphthalene | C14805 | Metabolism of xenobiotics by cytochrome P450(ko00980) |
| pos\_252 | pseudaminic acid | C20082 | Amino sugar and nucleotide sugar metabolism(ko00520);;Metabolic pathways(ko01100) |
| pos\_2541 | histidinal | C01929 | Metabolic pathways(ko01100);;Histidine metabolism(ko00340) |
| pos\_2548 | n-methylserotonin | C06212 | Tryptophan metabolism(ko00380) |
| pos\_2567 | carbamazepine | C06868 | Drug metabolism - cytochrome P450(ko00982) |
| pos\_2572 | phenylacetic acid | C07086 | Phenylalanine metabolism(ko00360);;Metabolic pathways(ko01100) |
| pos\_2575 | paromomycin | C00832 | Neomycin, kanamycin and gentamicin biosynthesis(ko00524) |
| pos\_2599 | n-acetylglutamic acid | C00624 | Metabolic pathways(ko01100);;Arginine biosynthesis(ko00220);;2-Oxocarboxylic acid metabolism(ko01210);;Biosynthesis of amino acids(ko01230) |
| pos\_2601 | nad | C00003 | Thiamine metabolism(ko00730);;Thermogenesis(ko04714);;Aldosterone synthesis and secretion(ko04925);;Calcium signaling pathway(ko04020);;Metabolic pathways(ko01100);;Longevity regulating pathway(ko04211);;Drug metabolism - other enzymes(ko00983);;Nicotinate and nicotinamide metabolism(ko00760);;Vitamin digestion and absorption(ko04977);;AMPK signaling pathway(ko04152);;Longevity regulating pathway - worm(ko04212);;Oxidative phosphorylation(ko00190) |
| pos\_2612 | thiosulfate | C00320 | Metabolic pathways(ko01100);;ABC transporters(ko02010);;Sulfur metabolism(ko00920) |
| pos\_2634 | 6-[2,3-dihydroxy-1-(hydroxymethyl)propyl]-1,2-dihydro-7-hydroxy-9-methoxy-cyclopenta[c][1]benzopyran-3,4-dione | C19590 | Metabolism of xenobiotics by cytochrome P450(ko00980) |
| pos\_2649 | chondroitin 4-sulfate | C00634 | Malaria(ko05144);;Glycosaminoglycan degradation(ko00531);;Metabolic pathways(ko01100) |
| pos\_267 | m-xylene | C07208 | Metabolic pathways(ko01100) |
| pos\_2687 | 4-fumarylacetoacetic acid | C01061 | Tyrosine metabolism(ko00350);;Metabolic pathways(ko01100) |
| pos\_2717 | dadp | C00206 | Metabolic pathways(ko01100);;Purine metabolism(ko00230) |
| pos\_272 | geranylhydroquinone | C10793 | Ubiquinone and other terpenoid-quinone biosynthesis(ko00130) |
| pos\_2727 | opc4-coa | C16335 | Metabolic pathways(ko01100);;alpha-Linolenic acid metabolism(ko00592) |
| pos\_2730 | histamine | C00388 | Fc epsilon RI signaling pathway(ko04664);;Gastric acid secretion(ko04971);;Histidine metabolism(ko00340);;Neuroactive ligand-receptor interaction(ko04080);;Asthma(ko05310);;Inflammatory mediator regulation of TRP channels(ko04750);;Synaptic vesicle cycle(ko04721);;Protein digestion and absorption(ko04974);;Metabolic pathways(ko01100) |
| pos\_2731 | 2-dehydro-d-gluconate | C06473 | Metabolic pathways(ko01100);;Pentose phosphate pathway(ko00030) |
| pos\_2734 | l-histidinol phosphate | C01100 | Metabolic pathways(ko01100);;Histidine metabolism(ko00340);;Biosynthesis of amino acids(ko01230) |
| pos\_2736 | 3-deoxy-d-manno-octulosonate 8-phosphate | C04478 | Metabolic pathways(ko01100) |
| pos\_2744 | l-palmitoylcarnitine | C02990 | Fatty acid degradation(ko00071);;Fatty acid metabolism(ko01212) |
| pos\_2759 | l-acetylcarnitine | C02571 | Insulin resistance(ko04931) |
| pos\_2768 | n-(5-phospho-d-ribosyl)anthranilate | C04302 | Biosynthesis of amino acids(ko01230);;Metabolic pathways(ko01100);;Phenylalanine, tyrosine and tryptophan biosynthesis(ko00400) |
| pos\_2769 | pc(16:1(9z)/0:0) | C04230 | Glycerophospholipid metabolism(ko00564);;Choline metabolism in cancer(ko05231) |
| pos\_2785 | raffinose | C00492 | ABC transporters(ko02010);;Galactose metabolism(ko00052);;Metabolic pathways(ko01100) |
| pos\_2788 | 2-phenylethanol | C05853 | Phenylalanine metabolism(ko00360) |
| pos\_2792 | 2'-deoxycytidine | C00881 | Metabolic pathways(ko01100);;Pyrimidine metabolism(ko00240);;ABC transporters(ko02010) |
| pos\_2802 | ubiquinone q1 | C00399 | Metabolic pathways(ko01100);;Ubiquinone and other terpenoid-quinone biosynthesis(ko00130);;Oxidative phosphorylation(ko00190) |
| pos\_2805 | udp-n-acetylmuramoyl-l-alanyl-d-gamma-glutamyl-meso-2,6-diaminopimelate | C04877 | Metabolic pathways(ko01100);;Lysine biosynthesis(ko00300) |
| pos\_2812 | stearidonic acid | C16300 | alpha-Linolenic acid metabolism(ko00592) |
| pos\_2843 | d-dethiobiotin | C01909 | Biotin metabolism(ko00780);;Metabolic pathways(ko01100) |
| pos\_2844 | 6-aminocaproic acid | C02378 | Metabolic pathways(ko01100) |
| pos\_2850 | sinapyl alcohol | C02325 | Metabolic pathways(ko01100) |
| pos\_2853 | alpha-tocotrienol | C14153 | Ubiquinone and other terpenoid-quinone biosynthesis(ko00130);;Metabolic pathways(ko01100) |
| pos\_2854 | fexofenadine | C06999 | Bile secretion(ko04976) |
| pos\_3 | cholic acid | C00695 | Primary bile acid biosynthesis(ko00120);;Bile secretion(ko04976);;Metabolic pathways(ko01100) |
| pos\_326 | 1-naphthol | C11714 | Metabolism of xenobiotics by cytochrome P450(ko00980) |
| pos\_331 | pe(22:2(13z,16z)/18:4(6z,9z,12z,15z)) | C00350 | Autophagy - animal(ko04140);;Glycerophospholipid metabolism(ko00564);;Kaposi sarcoma-associated herpesvirus infection(ko05167);;Autophagy - other(ko04136);;Retrograde endocannabinoid signaling(ko04723);;Pathogenic Escherichia coli infection(ko05130);;Glycosylphosphatidylinositol (GPI)-anchor biosynthesis(ko00563);;Metabolic pathways(ko01100) |
| pos\_34 | p-cymene | C06575 | Metabolic pathways(ko01100) |
| pos\_36 | phenol | C00146 | Tyrosine metabolism(ko00350);;Metabolic pathways(ko01100);;Protein digestion and absorption(ko04974) |
| pos\_362 | capsaicin | C06866 | Metabolic pathways(ko01100);;Inflammatory mediator regulation of TRP channels(ko04750) |
| pos\_388 | demethylphylloquinone | C13309 | Metabolic pathways(ko01100);;Ubiquinone and other terpenoid-quinone biosynthesis(ko00130) |
| pos\_39 | choline | C00114 | Bile secretion(ko04976);;ABC transporters(ko02010);;Metabolic pathways(ko01100);;Glycerophospholipid metabolism(ko00564);;Choline metabolism in cancer(ko05231);;Cholinergic synapse(ko04725);;Glycine, serine and threonine metabolism(ko00260) |
| pos\_395 | s-[2-(n7-guanyl)ethyl]-n-acetyl-l-cysteine | C14877 | Metabolism of xenobiotics by cytochrome P450(ko00980) |
| pos\_4 | l-proline | C00148 | Aminoacyl-tRNA biosynthesis(ko00970);;ABC transporters(ko02010);;Mineral absorption(ko04978);;Arginine and proline metabolism(ko00330);;Central carbon metabolism in cancer(ko05230);;Biosynthesis of amino acids(ko01230);;Metabolic pathways(ko01100);;Protein digestion and absorption(ko04974) |
| pos\_404 | 9(s)-hete | C14779 | Arachidonic acid metabolism(ko00590) |
| pos\_431 | ppa | C00254 | Biosynthesis of amino acids(ko01230);;Phosphonate and phosphinate metabolism(ko00440);;Phenylalanine, tyrosine and tryptophan biosynthesis(ko00400);;Metabolic pathways(ko01100) |
| pos\_457 | 2-oxosuberate | C16589 | Metabolic pathways(ko01100);;2-Oxocarboxylic acid metabolism(ko01210) |
| pos\_463 | jasmonic acid | C08491 | Metabolic pathways(ko01100);;alpha-Linolenic acid metabolism(ko00592) |
| pos\_474 | (3s,6e)-nerolidol | C09704 | Metabolic pathways(ko01100) |
| pos\_477 | n-glycolylneuraminic acid | C03410 | Amino sugar and nucleotide sugar metabolism(ko00520) |
| pos\_478 | caprylic acid | C06423 | Lipoic acid metabolism(ko00785);;Fatty acid biosynthesis(ko00061);;Metabolic pathways(ko01100) |
| pos\_486 | biochanin a | C00814 | Metabolic pathways(ko01100) |
| pos\_492 | phthalic acid | C01606 | Metabolic pathways(ko01100);;ABC transporters(ko02010) |
| pos\_499 | oleic acid | C00712 | Biosynthesis of unsaturated fatty acids(ko01040);;Longevity regulating pathway - worm(ko04212);;Fatty acid biosynthesis(ko00061) |
| pos\_5 | citric acid | C00158 | Biosynthesis of amino acids(ko01230);;Central carbon metabolism in cancer(ko05230);;Taste transduction(ko04742);;Metabolic pathways(ko01100);;Alanine, aspartate and glutamate metabolism(ko00250);;Glyoxylate and dicarboxylate metabolism(ko00630);;Carbon metabolism(ko01200);;2-Oxocarboxylic acid metabolism(ko01210);;Glucagon signaling pathway(ko04922);;Citrate cycle (TCA cycle)(ko00020) |
| pos\_507 | androstanedione | C00674 | Steroid hormone biosynthesis(ko00140) |
| pos\_52 | palmitoyl glucuronide | C03033 | Bile secretion(ko04976);;Pentose and glucuronate interconversions(ko00040);;Metabolic pathways(ko01100) |
| pos\_522 | dihomo-linoleate (20:2n6) | C16525 | Biosynthesis of unsaturated fatty acids(ko01040) |
| pos\_529 | 5-aminopentanal | C12455 | Lysine degradation(ko00310);;Metabolic pathways(ko01100) |
| pos\_53 | toluene | C01455 | Metabolic pathways(ko01100) |
| pos\_545 | nadph | C00006 | Aldosterone synthesis and secretion(ko04925);;Metabolic pathways(ko01100);;Thyroid hormone synthesis(ko04918);;Glutathione metabolism(ko00480);;Drug metabolism - other enzymes(ko00983);;Nicotinate and nicotinamide metabolism(ko00760) |
| pos\_556 | 5-hydroxylysine | C16741 | Metabolic pathways(ko01100);;Lysine degradation(ko00310) |
| pos\_560 | dodecanedioic acid | C16308 | alpha-Linolenic acid metabolism(ko00592) |
| pos\_564 | 5-hydroxyisourate | C11821 | Metabolic pathways(ko01100);;Purine metabolism(ko00230) |
| pos\_57 | 7-dehydrocholesterol | C05443 | Rheumatoid arthritis(ko05323);;Insect hormone biosynthesis(ko00981);;Vitamin digestion and absorption(ko04977);;Steroid biosynthesis(ko00100);;Metabolic pathways(ko01100) |
| pos\_577 | prostaglandin j2 | C05957 | Metabolic pathways(ko01100);;Serotonergic synapse(ko04726);;Arachidonic acid metabolism(ko00590) |
| pos\_597 | heteroxanthin | C16353 | Metabolic pathways(ko01100);;Caffeine metabolism(ko00232) |
| pos\_602 | 2'-deoxyinosine triphosphate | C01345 | Purine metabolism(ko00230) |
| pos\_606 | 6-deoxocastasterone | C15802 | Metabolic pathways(ko01100) |
| pos\_607 | 11-dehydro-thromboxane b2 | C05964 | Serotonergic synapse(ko04726);;Arachidonic acid metabolism(ko00590);;Metabolic pathways(ko01100) |
| pos\_61 | 5-aminoimidazole | C05239 | Metabolic pathways(ko01100);;Purine metabolism(ko00230) |
| pos\_614 | fumaric acid | C00122 | Citrate cycle (TCA cycle)(ko00020);;Arginine biosynthesis(ko00220);;Butanoate metabolism(ko00650);;Glucagon signaling pathway(ko04922);;Tyrosine metabolism(ko00350);;Renal cell carcinoma(ko05211);;Oxidative phosphorylation(ko00190);;Pyruvate metabolism(ko00620);;Carbon metabolism(ko01200);;Alanine, aspartate and glutamate metabolism(ko00250);;Metabolic pathways(ko01100);;Phenylalanine metabolism(ko00360);;Central carbon metabolism in cancer(ko05230);;Nicotinate and nicotinamide metabolism(ko00760);;Pathways in cancer(ko05200) |
| pos\_639 | 4-hydroxybenzoic acid | C00156 | Ubiquinone and other terpenoid-quinone biosynthesis(ko00130);;Folate biosynthesis(ko00790);;Metabolic pathways(ko01100) |
| pos\_65 | o-xylene | C07212 | Metabolic pathways(ko01100) |
| pos\_650 | 4-hydroxyproline | C01157 | ABC transporters(ko02010);;Arginine and proline metabolism(ko00330);;Metabolic pathways(ko01100) |
| pos\_659 | stearidonic acid | C16300 | alpha-Linolenic acid metabolism(ko00592) |
| pos\_662 | all-trans-retinoic acid | C00777 | Metabolic pathways(ko01100);;Pathways in cancer(ko05200);;Non-small cell lung cancer(ko05223);;Th17 cell differentiation(ko04659);;Gastric cancer(ko05226);;Retinol metabolism(ko00830);;Intestinal immune network for IgA production(ko04672);;Small cell lung cancer(ko05222) |
| pos\_663 | myristic acid | C06424 | Metabolic pathways(ko01100);;Fatty acid biosynthesis(ko00061) |
| pos\_671 | l-methionine | C00073 | Protein digestion and absorption(ko04974);;Metabolic pathways(ko01100);;Central carbon metabolism in cancer(ko05230);;Biosynthesis of amino acids(ko01230);;Mineral absorption(ko04978);;Antifolate resistance(ko01523);;2-Oxocarboxylic acid metabolism(ko01210);;Cysteine and methionine metabolism(ko00270);;Aminoacyl-tRNA biosynthesis(ko00970) |
| pos\_683 | linamarin | C01594 | Metabolic pathways(ko01100) |
| pos\_695 | gamma-linolenic acid | C06426 | Linoleic acid metabolism(ko00591);;Metabolic pathways(ko01100);;Biosynthesis of unsaturated fatty acids(ko01040) |
| pos\_709 | 5-aminopentanoic acid | C00431 | Lysine degradation(ko00310);;Arginine and proline metabolism(ko00330);;Metabolic pathways(ko01100) |
| pos\_717 | 2-hydroxybutyric acid | C05984 | Propanoate metabolism(ko00640) |
| pos\_722 | eicosenoic acid | C16526 | Biosynthesis of unsaturated fatty acids(ko01040) |
| pos\_723 | leukotriene a4 | C00909 | Arachidonic acid metabolism(ko00590);;Serotonergic synapse(ko04726);;Metabolic pathways(ko01100) |
| pos\_730 | ce(22:6(4z,7z,10z,13z,16z,19z)) | C02530 | Fat digestion and absorption(ko04975);;Ovarian steroidogenesis(ko04913);;Bile secretion(ko04976);;Cholesterol metabolism(ko04979);;Steroid biosynthesis(ko00100);;Vitamin digestion and absorption(ko04977) |
| pos\_735 | abietinol | C11882 | Metabolic pathways(ko01100) |
| pos\_743 | crustecdysone | C02633 | Insect hormone biosynthesis(ko00981) |
| pos\_75 | (r)-4-phosphopantoate | C18911 | Metabolic pathways(ko01100);;Pantothenate and CoA biosynthesis(ko00770) |
| pos\_752 | 3-methoxy-4-hydroxyphenylglycol glucuronide | C03033 | Pentose and glucuronate interconversions(ko00040);;Bile secretion(ko04976);;Metabolic pathways(ko01100) |
| pos\_754 | biocytin | C05552 | Vitamin digestion and absorption(ko04977);;Biotin metabolism(ko00780);;Metabolic pathways(ko01100) |
| pos\_759 | 5-l-glutamyl-l-alanine | C03740 | Glutathione metabolism(ko00480);;Metabolic pathways(ko01100) |
| pos\_766 | l-arginine | C00062 | Amoebiasis(ko05146);;Aminoacyl-tRNA biosynthesis(ko00970);;ABC transporters(ko02010);;Arginine and proline metabolism(ko00330);;Chagas disease(ko05142);;D-Arginine and D-ornithine metabolism(ko00472);;Arginine biosynthesis(ko00220);;Central carbon metabolism in cancer(ko05230);;Monobactam biosynthesis(ko00261);;mTOR signaling pathway(ko04150);;Biosynthesis of amino acids(ko01230);;Amyotrophic lateral sclerosis(ko05014);;Metabolic pathways(ko01100);;Protein digestion and absorption(ko04974) |
| pos\_784 | 4-imidazolone-5-propionic acid | C03680 | Metabolic pathways(ko01100);;Histidine metabolism(ko00340) |
| pos\_805 | capric acid | C01571 | Metabolic pathways(ko01100) |
| pos\_806 | norfloxacin | C06687 | ABC transporters(ko02010) |
| pos\_809 | hexadecanedioic acid | C19615 | Metabolic pathways(ko01100) |
| pos\_813 | phytosphingosine | C12144 | Sphingolipid metabolism(ko00600);;Metabolic pathways(ko01100) |
| pos\_816 | imidazoleacetic acid ribotide | C04437 | Histidine metabolism(ko00340) |
| pos\_819 | endomorphin-2 | C15891 | Neuroactive ligand-receptor interaction(ko04080) |
| pos\_83 | 8z,11z,14z-heptadecatrienoic acid | C16344 | alpha-Linolenic acid metabolism(ko00592) |
| pos\_835 | 3-dehydroxycarnitine | C01181 | Lysine degradation(ko00310);;ABC transporters(ko02010);;Metabolic pathways(ko01100) |
| pos\_856 | n-ethyl trans-2-cis-6-nonadienamide | C06551 | Metabolic pathways(ko01100) |
| pos\_862 | l-lysine | C00047 | 2-Oxocarboxylic acid metabolism(ko01210);;ABC transporters(ko02010);;Aminoacyl-tRNA biosynthesis(ko00970);;Lysine degradation(ko00310);;Lysine biosynthesis(ko00300);;Biosynthesis of amino acids(ko01230);;Protein digestion and absorption(ko04974);;Metabolic pathways(ko01100);;Biotin metabolism(ko00780) |
| pos\_869 | benzene | C01407 | Metabolic pathways(ko01100) |
| pos\_871 | chenodeoxycholic acid glycine conjugate | C05466 | Cholesterol metabolism(ko04979) |
| pos\_879 | acetylcholine | C01996 | Taste transduction(ko04742);;Pancreatic secretion(ko04972);;Regulation of actin cytoskeleton(ko04810);;Synaptic vesicle cycle(ko04721);;Cholinergic synapse(ko04725);;Salivary secretion(ko04970);;Nicotine addiction(ko05033);;Bile secretion(ko04976);;Gastric acid secretion(ko04971);;Insulin secretion(ko04911);;Neuroactive ligand-receptor interaction(ko04080);;Glycerophospholipid metabolism(ko00564);;cAMP signaling pathway(ko04024) |
| pos\_884 | anandamide | C11695 | Inflammatory mediator regulation of TRP channels(ko04750);;Thermogenesis(ko04714);;Retrograde endocannabinoid signaling(ko04723);;Neuroactive ligand-receptor interaction(ko04080);;Axon regeneration(ko04361) |
| pos\_9 | retinoic acid | C00777 | Metabolic pathways(ko01100);;Pathways in cancer(ko05200);;PPAR signaling pathway(ko03320);;Non-small cell lung cancer(ko05223);;Th17 cell differentiation(ko04659);;Adipocytokine signaling pathway(ko04920);;Gastric cancer(ko05226);;Intestinal immune network for IgA production(ko04672);;Retinol metabolism(ko00830);;Small cell lung cancer(ko05222) |
| pos\_911 | 4-pyridoxic acid | C00847 | Metabolic pathways(ko01100);;Vitamin B6 metabolism(ko00750) |
| pos\_913 | spermine | C00750 | beta-Alanine metabolism(ko00410);;Arginine and proline metabolism(ko00330);;Metabolic pathways(ko01100);;Glutathione metabolism(ko00480);;Pantothenate and CoA biosynthesis(ko00770);;Bile secretion(ko04976) |
| pos\_919 | 4-hydroxyandrostenedione glucuronide | C11455 | Metabolic pathways(ko01100);;Steroid biosynthesis(ko00100) |
| pos\_92 | adenine | C00147 | Purine metabolism(ko00230);;Metabolic pathways(ko01100) |
| pos\_975 | carnosine | C00386 | Metabolic pathways(ko01100);;beta-Alanine metabolism(ko00410);;Histidine metabolism(ko00340) |
| pos\_980 | n-carbamoylputrescine | C00436 | Metabolic pathways(ko01100);;Arginine and proline metabolism(ko00330) |
